# AMPK signaling in osteoarthritis: from mechanisms to targeted therapeutics

**DOI:** 10.3389/fphar.2025.1681610

**Published:** 2025-11-07

**Authors:** Lin Chen, Xiu-Hua Hu, Xin-Yi Wu, Xin Zhang, Yu-Xin Han, Yi Liu, Guang-Yao Chen, Qing-Wen Tao

**Affiliations:** 1 Graduate school, Beijing University of Chinese Medicine, Beijing, China; 2 School of Life Sciences, Beijing University of Chinese Medicine, Beijing, China; 3 Traditional Chinese Medicine Department of Rheumatism, China-Japan Friendship Hospital, Beijing, China

**Keywords:** osteoarthritis, AMPK signaling, targeted-therapeutics, molecular mechanisms, metabolic disorder

## Abstract

Osteoarthritis (OA) is a common degenerative joint disease characterized by joint pain, swelling, stiffness, and limited mobility. Current treatments primarily offer partial and short-term relief, with concerns about the potential side effects. This underscores the need for safer and more effective therapeutic strategies. AMP-activated protein kinase (AMPK), a key regulator of cellular energy metabolism, plays an essential role in maintaining the homeostasis of articular cartilage, synovium, and subchondral bone. AMPK signaling has been shown to protect joint tissues from damage caused by mechanical stress and inflammatory responses. Studies suggest that modulating AMPK signaling can influence processes such as autophagy, inflammation, and oxidative stress through downstream targets, including the SIRT family and FoxO family. These mechanisms may help reduce cartilage degradation, osteophyte formation, and synovial inflammation. This review provides a systematic overview of the role of AMPK signaling in joint tissues and explores its potential as a therapeutic target for OA, with the aim of informing the development of targeted therapies that may contribute to more effective and safer management of OA symptoms.

## 1 Introduction

Osteoarthritis (OA) is one of the most common arthritis, characterized by cartilage degeneration, bone remodeling, osteophyte formation, and synovial inflammation, resulting in joint pain, swelling, stiffness, and a loss of normal joint function (Kolasinski et al., 2020). An epidemiology study found that approximately 300 million people worldwide suffer from osteoarthritis, which results in huge medical expenses and indirect costs due to reduced mobility and motor function (GBD, 2017 Disease and Injury Incidence and Prevalence Collaborators, 2018). The contemporary management of OA primarily emphasizes a multifaceted approach that includes structured exercise regimens, analgesics for pain relief, the application of non-steroidal and anti-inflammatory drugs, and, in cases of advanced degeneration, joint replacement surgery (Bijlsma et al., 2011; Katz et al., 2021). However, there are many concerns regarding the adverse reactions of analgesics and non-steroidal anti-inflammatory drugs (NSAIDs), as well as the prognosis following surgical interventions, which necessitate novel therapies to improve symptoms and ensure patient safety (Guermazi et al., 2020; Price et al., 2018; Volkow and McLellan, 2016). The understanding of the pathological mechanisms underlying OA continues to evolve. Growing evidence now emphasizes the critical role of the cellular signaling pathways, particularly those involving inflammatory mediators, metabolic intermediates that are considered key drivers in the pathophysiology of OA (Hunter and Bierma-Zeinstra, 2019). In this case, the therapies aimed at modulating OA-associated pathways to maintain joint homeostasis may be a pivotal measure to reduce the symptoms and prevent the progression of OA (Yao et al., 2023). As a primary sensor of cellular energy, AMP-activated protein kinase (AMPK) plays an important role in maintaining cell and tissue homeostasis by regulating metabolism in response to fluctuations in the ratio of ADP/ATP or AMP/ATP (Lin and Hardie, 2018). Interestingly, recent research indicates that AMPK not only indirectly affects OA through metabolic disorders, which contribute to obesity and subsequent biomechanical stress on joints, but also directly participates in OA-related pathways to protect cells and tissues of the joint from stress-induced damage while mitigating inflammation (Liu-Bryan, 2015). Herein, we explore the recent advances in understanding the role of AMPK in regulating joint tissue homeostasis, as well as its potential as a therapeutic target in the treatment of OA.

## 2 AMPK signaling

AMPK is a heterotrimeric complex composed of α-subunits with a catalytic domain and regulatory β- and γ-subunits (Calabrese et al., 2014). The α-subunit contains a kinase domain and an activation loop, Thr172, which is phosphorylated by an upstream kinase to achieve full activation of AMPK (Oakhill et al., 2012). The γ-subunit enables AMPK to sense cellular energy levels and responses to fluctuations in the ATP-to-AMP or ATP-to-ADP ratio, facilitating AMPK phosphorylation by upstream kinases (Garcia and Shaw, 2017). Three subunits of AMPK have different isoforms that can form various αβγ complexes and are found in different types of cells. In chondrocytes, the α1, α2, β1, and β2 subunits, as well as the γ1 subunit, are present (Terkeltaub et al., 2011). In subchondral bone, mice lacking AMPKα1 or AMPKα2 presented reduced bone mass. Specifically, AMPKα1 inhibits the receptor activator of nuclear factor κB (RANK) signaling in osteoclast precursors, leading to the downregulation of osteoclast differentiation (Kang H. et al., 2013), whereas AMPKα2 exhibits increased osteogenesis (Wang Y. G. et al., 2016a). The β-subunits are also associated with bone mass and microstructure; however, the deficiency of β-subunits has no effect on the number of osteoclasts or osteoblasts. The underlying mechanism needs to be further explored (Quinn et al., 2010). It is worth noting that the β2 isoform is highly expressed in skeletal muscle and is associated with glucose uptake and fatty acid oxidation, which is important for the regulation of AMPK in the metabolism of glucose and lipid (Steinberg et al., 2010). The structure and precise function of each subunit have not yet been fully confirmed, and the mechanisms explaining the activation of AMPK are still being investigated. It is now believed that AMP promotes Thr172 phosphorylation by upstream kinases, with liver kinase B1 (LKB1) being the main kinase responsible (Woods et al., 2003). Calmodulin-dependent protein kinase kinases (CAMKKs), including CAMKKα and CAMKKβ, have also been shown to phosphorylate AMPK in response to decreased ATP levels caused by ATP-driven Ca^2+^ pumps facilitating Ca^2+^ entry into cells (Hurley et al., 2005).

**FIGURE 1:**
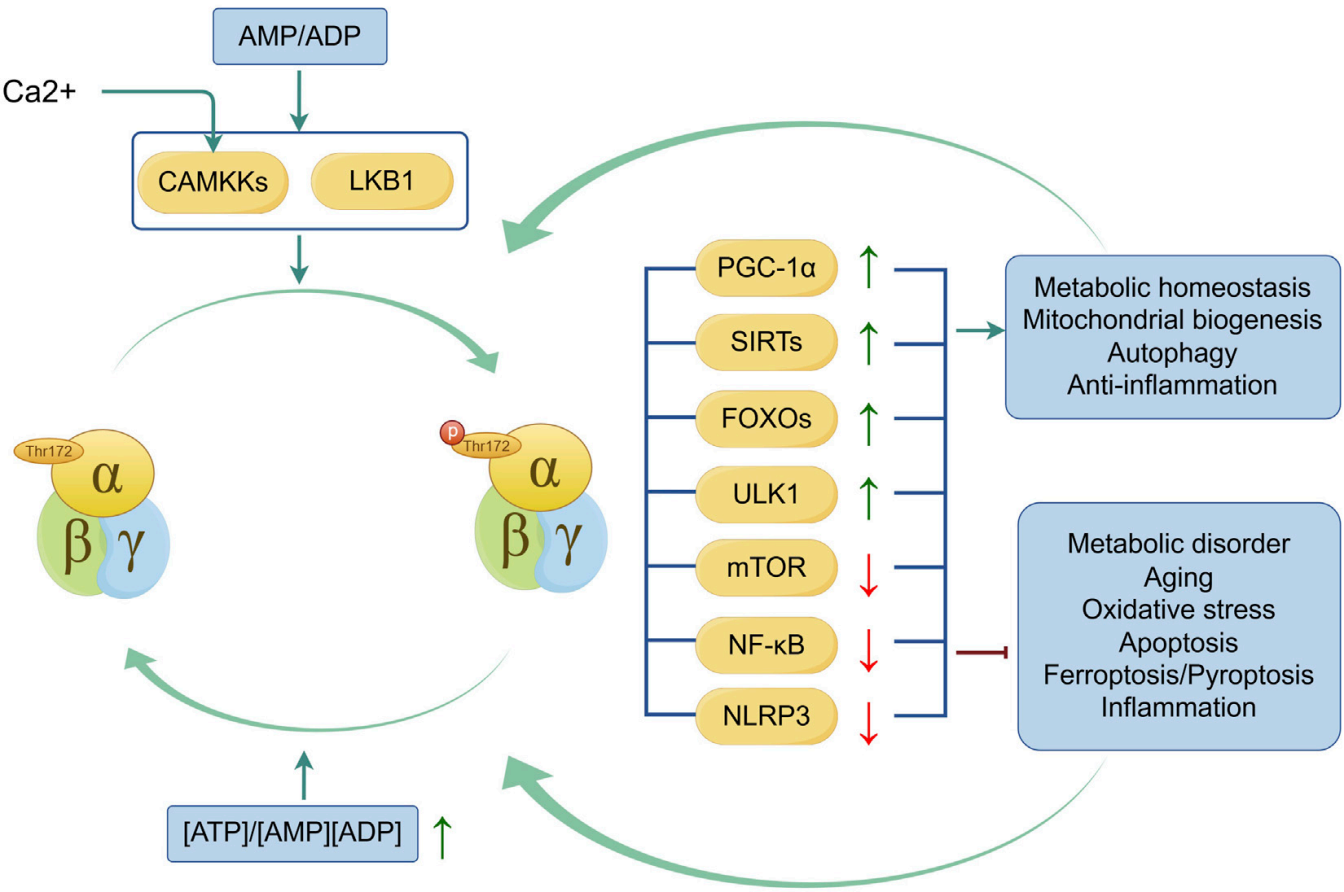
Mechanism and regulation of AMPK activation. LKB1 and CAMKKs (when facilitating Ca^2+^ entry into cells) are involved in the phosphorylation of AMPK, a process induced by the ATP-to-AMP or ATP-to-ADP ratio. Activation of AMPK and its downstream proteins of AMPK, including Peroxisome proliferator-activated receptor γ coactivator 1α (PGC-1α), Sirtuins(SIRTs), Forkhead box Os (FOXOs), Unc-51-like autophagy activating kinase 1(ULK1), mammalian target of rapamycin(mTOR), nuclear factor-kappa B(NF-κB) and Nod-like receptor protein 3 (NLRP3) improve metabolic homeostasis, mitochondrial biogenesis, autophagy and anti-inflammation and inhibit metabolic disorder, aging, oxidative stress, apoptosis, ferroptosis, pyroptosis and inflammation. Conversely, the activities improved by AMPK signaling help the phosphorylation of AMPK, and the inhibited activities suppress the activation of AMPK. The green line indicates promotion, and the red line indicates suppression. ↑ signifies an increase, while ↓ signifies a decrease.

As a conserved energy sensor, AMPK can respond swiftly to the ratio of ATP to AMP in cells and restore ATP levels during metabolic stress by inhibiting ATP-consuming biosynthesis pathways and activating ATP-generating catabolic pathways (Kahn et al., 2005). Moreover, AMPK activates a variety of downstream proteins and participates in multiple functional pathways, including autophagy, mitochondrial biogenesis, and inflammation signaling pathways. Also, activation of AMPK is influenced by these metabolic activities, so as to maintain metabolic homeostasis and improve cellular resistance to stress (Trefts and Shaw, 2021). Notably, these findings highlight the significant role of AMPK signaling in maintaining joint homeostasis (Figure 1).

## 3 AMPK signaling is involved in the homeostasis of the joint

**FIGURE 2:**
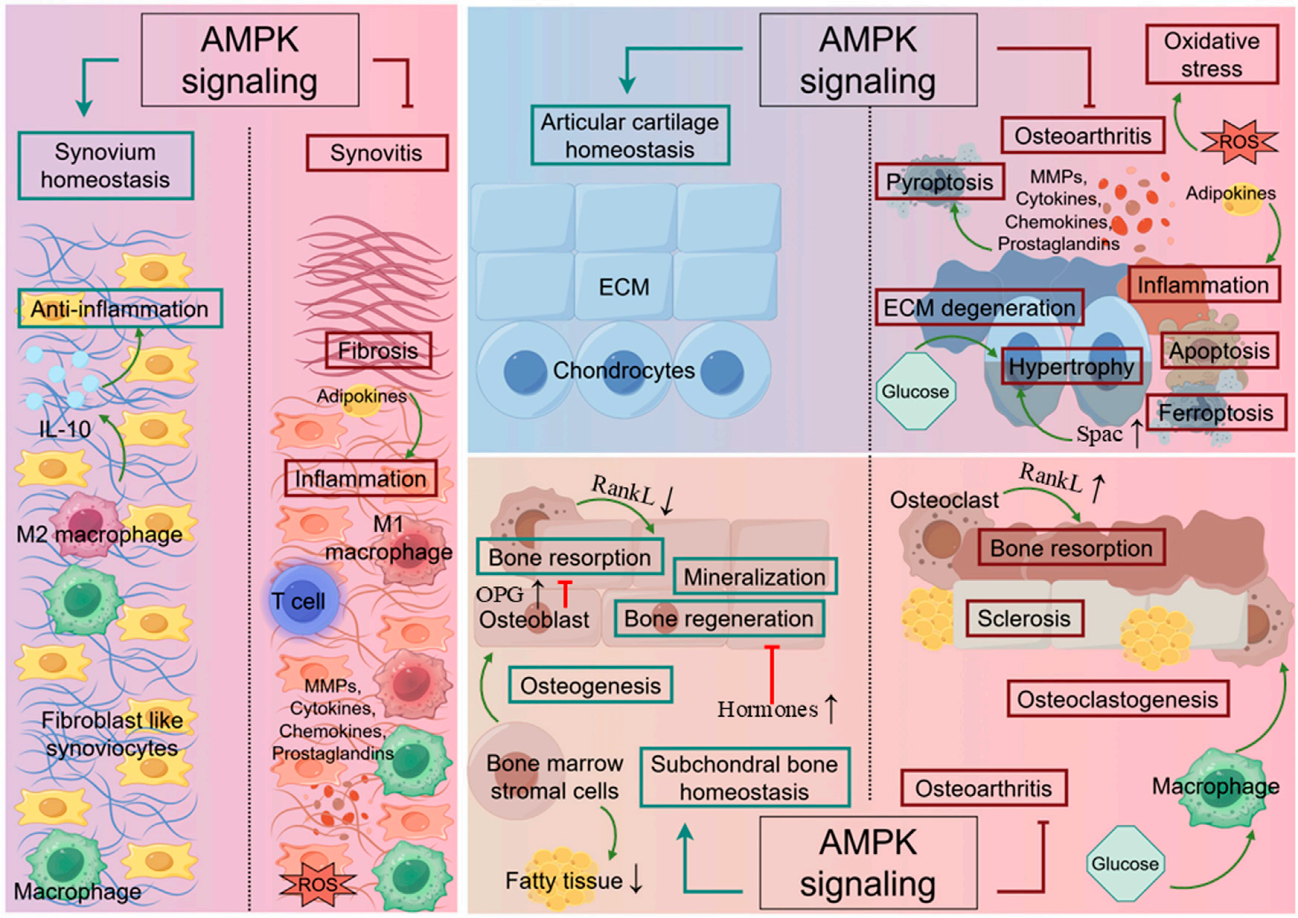
The role of AMPK in joint. In cartilage, AMPK signaling promotes chondrocyte proliferation and enhances ECM synthesis in response to mechanical loading. Simultaneously, it inhibits ECM degeneration, chondrocyte hypertrophy, apoptosis, ferroptosis, oxidative stress and inflammation triggered by MMPs, cytokines, chemokines, and prostaglandins. In synovium, AMPK signaling involves in preventing synovitis by suppressing the activation of inflammatory cells, pyroptosis and fibrosis. In subchondral bone, AMPK signaling facilitates bone regeneration by activating autophagy while preventing excessive bone resorption and sclerosis. The green line indicates promotion, and the red line indicates suppression. ↑ signifies an increase, while ↓ signifies a decrease.

Under physiological conditions, articular cartilage, which is composed of chondrocytes and an extracellular matrix (ECM), creates a smooth surface and absorbs external shock, assisting in the transmission of loads with low friction. In response to mechanical loading, chondrocytes promote their own proliferation and enhance ECM synthesis to maintain cartilage integrity (Salinas et al., 2019). The synovium produces synovial fluid to reduce friction and absorb shock, as well as to transport nutrients and waste in the joint (Levick and McDonald, 1995). Subchondral bone dynamically adapts to the mechanical forces exerted on the joint through a coordinated process of bone remodeling, thereby re-establishing normal physiological conditions (Stegen and Carmeliet, 2024). In OA, articular cartilage and the microstructure alteration of subchondral bone suffer from progressive thinning and fragmentation, resulting in increased friction between the two articular surfaces, along with inflammation (Neogi et al., 2009). Synovium, when it develops into synovitis, undergoes changes in the composition and volume of synovial fluid, leading to increased inflammation and pain sensitization (Benito et al., 2005). Researchers have realized that metabolic disorders, such as obesity, contribute not only to the mechanical load causing joint wear and tear but also to elevated metabolic factors, particularly altered levels of adipokines (Zhuo et al., 2012). This disruption affects the function of joint cells, resulting in the non-load-bearing joints in patients with metabolic syndrome suffering OA (Thijssen et al., 2015). In recent years, increasing studies have proved that AMPK, as a crucial regulator of energy metabolism, participates in joint dynamical adaptation (Liu-Bryan and Terkeltaub, 2015) (Figure 2). Typically, activation of AMPK promotes osteogenic differentiation and mineralization as well as suppresses osteoclasts and bone resorption by downregulating receptor activator of nuclear kappa B ligand (RANKL), which is an essential transcription factor for osteoclast differentiation (Lee et al., 2010; Kim J. Y. et al., 2015; Park S. H. et al., 2020; Kanazawa et al., 2008). AMPK has also been found to promote osteogenesis and inhibit adipogenesis in bone marrow stromal cells (BMSCs), which identifies the significance of AMPK signaling in fatty metabolism (Wang Y. G. et al., 2016b). Other important cellular activities involved in AMPK-mediated homeostasis in joints are listed below.

### 3.1 Metabolic homeostasis and disorder

**FIGURE 3:**
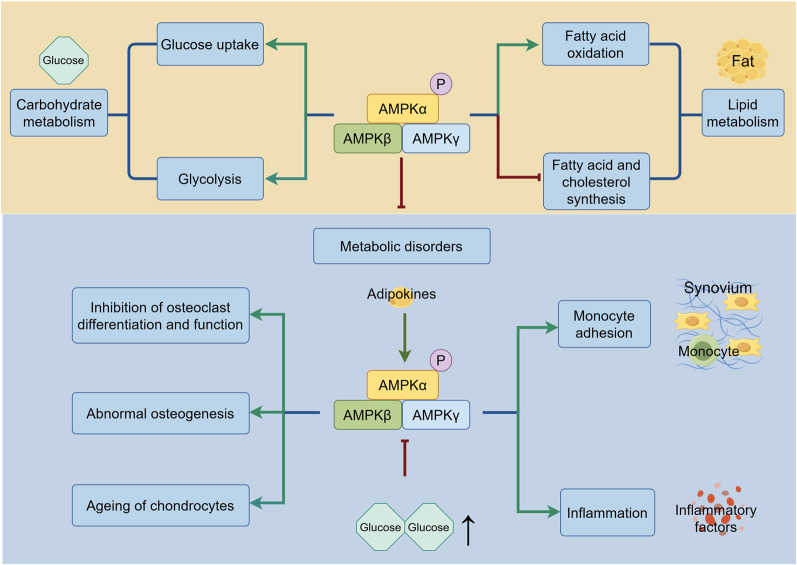
The regulation of AMPK in metabolism. Under the metabolism homeostasis, AMPK regulates carbohydrate metabolism by improving glucose uptake and glycolysis, and lipid metabolism by increasing fatty acid oxidation and lowering fatty acid and cholesterol synthesis. During the metabolic disorders, AMPK promotes monocyte adhesion and inflammation induced by adipokines. Suppression of AMPK caused by hyperglycemia leads to inhibition of osteoclast differentiation and function, abnormal osteogenesis and ageing of chondrocytes. The green line indicates promotion, and the red line indicates suppression.↑ signifies an increase.

Due to its heightened sensitivity to alterations in intracellular AMP levels, AMPK plays an important role in the metabolism of glucose and lipid to regulate homeostasis of joints (Figure 3). On the one hand, AMPK improves metabolic disorders and inhibits the development of obesity, which is one of the risk factors for OA. It is noted that AMPK activity is lower in multiple tissues of individuals with obesity and insulin resistance (Bandyopadhyay et al., 2006). Moreover, a great number of studies show that AMPK activation reduces lipid storage by promoting fatty acid oxidation while suppressing fatty acid and cholesterol synthesis, regulates carbohydrate metabolism by increasing glucose uptake or promoting glycolysis, thus avoiding obese diseases that cause wear and tear of joints (Steinberg and Carling, 2019). The excellent effect of AMPK in improving metabolic disorders attracts researchers to develop AMPK activators for application in metabolic diseases, as detailed in part 5.2. On the other hand, researchers have also realized that metabolic disorders contribute to elevated metabolic factors; this disruption results in the non-load-bearing joints in patients with metabolic syndrome suffering OA. In lipid metabolism, adipokines deserve attention (Zhuo et al., 2012). Studies have shown that adipokine is significantly increased in synovial cells from OA patients, alongside the raised expression of inflammatory factors and monocyte adhesion via activation of AMPK in human synovial fibroblasts (Tang et al., 2007; Law et al., 2020). These studies considered the pro-inflammatory effect of AMPK regulated by adipokines. However, most studies revealed the anti-inflammatory effect of AMPK, which are discussed in part 3.4. In glucose metabolism, hyperglycemia inhibits the AMPK signaling pathway, leading to the suppression of osteoclast differentiation and function, abnormal osteogenesis and the accelerated ageing of chondrocytes by increasing ROS and inhibiting autophagy (Zhen et al., 2010; Wang B. et al., 2021; Cai et al., 2018). Besides, AMPK activity decreases with age, resulting in suppressed insulin-stimulated glucose uptake and ultimately leading to bone metabolism disorders and OA. The exact mechanisms by which AMPK activity is diminished in ageing tissues are not fully understood; however, one study showed that increased DNA-dependent protein kinase (DNA-PK), which is related to increased DNA double-strand breaks, was responsible for decreased AMPK activity in skeletal muscle (Park et al., 2017).

The relationship between metabolic disorders and OA is not only related to abnormal glucose and lipid levels, but is also characterized by oxidative stress, autophagy, and inflammation. The following discussion covers these topics.

### 3.2 Mitochondrial biogenesis and oxidative stress

**FIGURE 4:**
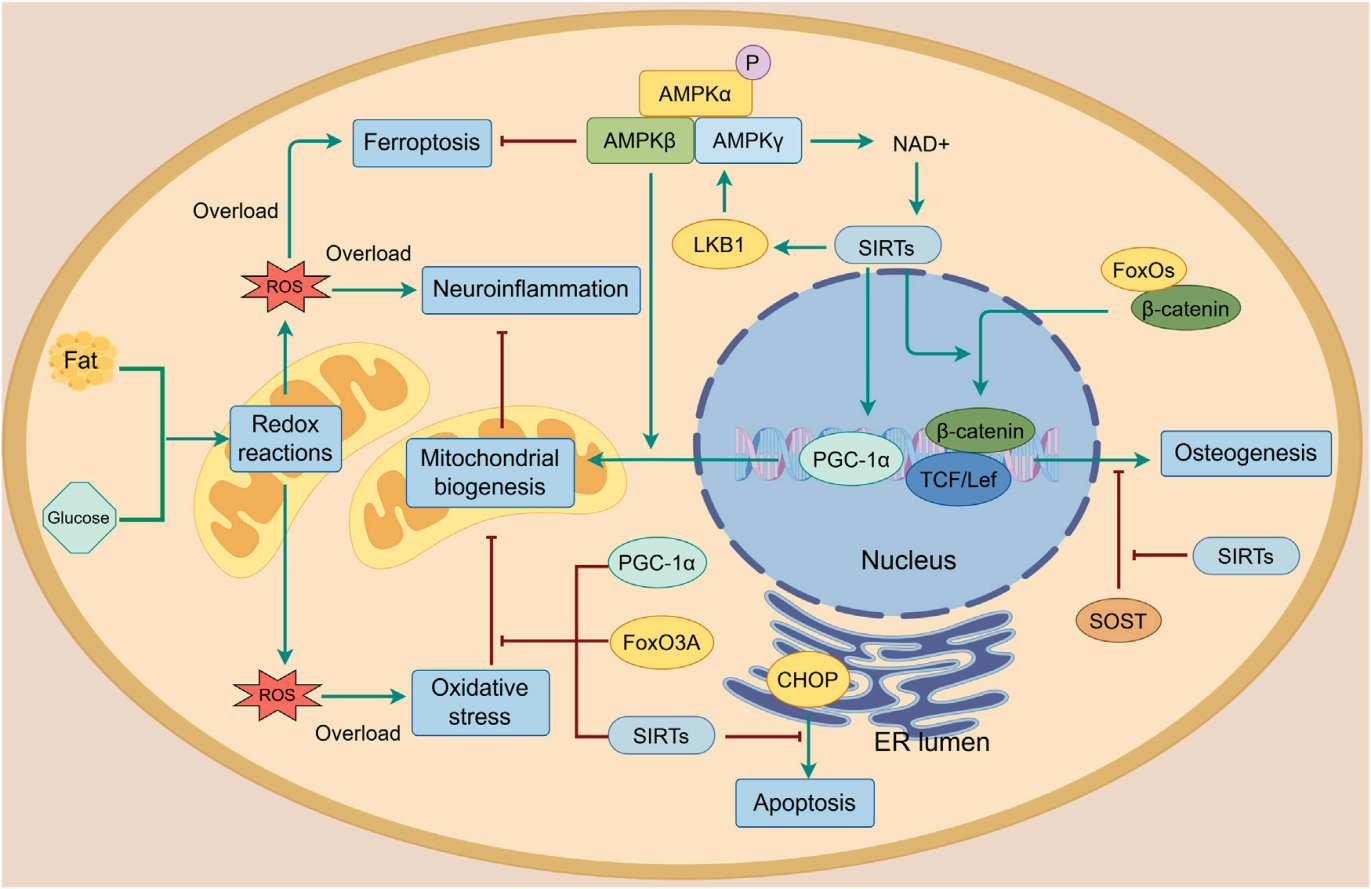
Mitochondrial biogenesis and oxidative stress in the joint. Reactive oxygen species (ROS) are produced during lipid and glucose metabolism. When there is an overload, mitochondrial biogenesis is damaged, resulting in oxidative stress, ferroptosis, neuroinflammation and apoptosis. AMPK and its downstream targets including PGC-1α, SIRTs and FoxO3A protect mitochondrial biogenesis from oxidative stress and enhance it. AMPK activates SIRTs by increasing cellular NAD^+^ levels. SIRTs can also phosphorylate AMPK through LKB1 to form a positive feedback loop that improves mitochondrial biogenesis. Additionally, the AMPK-SIRT signaling pathway inhibits apoptosis by reducing CHOP levels and improves osteogenesis by protecting β-catenin from sclerostin (SOST) and FoxOs. The green line indicates promotion, and the red line indicates suppression.

During the development of OA, mitochondrial dysfunction causes a relative overload of reactive oxygen species (ROS), including nitric oxide, superoxide anion and hydrogen peroxide, leading to oxidative stress and resultant metabolic disorder (Lepetsos and Papavassiliou, 2016). During these processes, AMPK and its downstream signaling attempt to maintain the homeostasis of mitochondrial biogenesis and reduction-oxidation (redox) reactions (Figure 4). Mitochondrial biogenesis regulator peroxisome proliferator-activated receptor γ coactivator 1α (PGC-1α) is the key participant and is essential for AMPK-mediated mitochondrial biogenesis (O’Neill et al., 2013). The deletion of either AMPK or PGC-1α leads to a decrease in mitochondrial proteins with impaired mitochondria and increased ROS, resulting in metabolic disorder. However, the mechanism by which the two interact with each other is not fully understood (Leick et al., 2010; O’Neill et al., 2011). Sirtuin (SIRT), an energy sensor that shares similar effects on diverse processes with AMPK, is also important in AMPK-mediated mitochondrial biogenesis (Wang Y. et al., 2015). On one hand, AMPK phosphorylation increases cellular NAD^+^ level, which boosts SIRT activity and helps protect mitochondrial homeostasis of chondrocytes from oxidative stress by clearing ROS that damages mitochondrial DNA (Cantó et al., 2009; Chen L. Y. et al., 2018). On the other hand, SIRT deacetylates LKB1 and thereby increases the activation of AMPK, accomplishing positive feedback regulation (Chen Y. et al., 2021; Ruderman et al., 2010). They work together to maintain joint homeostasis. In the cartilage, abnormal biomechanical loading or other environmental stress causes an excessive unfolded protein response (UPR) within the endoplasmic reticulum (ER) lumen. This is highly associated with inflammation and induces apoptosis by activating C/EBP homologous protein (CHOP), while the activation of AMPK and SIRT reduces excessive CHOP expression when chondrocytes are subjected to biomechanical injury (Zhang and Kaufman, 2008; Kang X. et al., 2018). Furthermore, SIRT is involved in the regulation of the AMPK-PGC-1α axis, which protects chondrocytes from impairments caused by abnormal catabolic responses by enhancing mitochondrial biogenesis (Wang Y. et al., 2015). Regulations of this axis in the spinal cord have been reported to alleviate OA pain caused by neuroinflammation (Sun J. et al., 2022). It should be noted that the activity of AMPK, SIRTs and PGC-1α is reduced by metabolic disturbances. For instance, an elevated homocysteine level induces mitochondrial dysfunction and stimulates oxidative stress, thereby disturbing chondrocyte metabolism and decreasing the levels of AMPK, SIRTs and PGC-1α proteins in OA chondrocytes (Ma et al., 2018). Ferroptosis, an iron-dependent form of non-apoptotic cell death, is one of the outcomes of ROS overload and is involved in the development of OA (Dixon et al., 2012; Stockwell et al., 2017; An et al., 2023). Studies have shown that AMPK signaling is necessary for the inhibition of ferroptosis through the inhibitory phosphorylation of acetyl-CoA carboxylase 1 (ACC1) and other probable substrates required for lipid biosynthesis. This protects cells from the accumulation of lipid hydroperoxides and ferroptosis in chondrocytes, thereby alleviating OA (Li C. et al., 2020; Zou et al., 2025; Xie et al., 2023). Forkhead box O3A (FoxO3A) is another factor involved in AMPK-mediated mitochondrial biogenesis that has the same ability as PGC-1α in limiting oxidative stress (Zhao et al., 2014). Damage to the activity and sensitivity of AMPK, SIRTs, PGC-1α and FoxOs is also evident in cartilage with age. Researchers considered that dysregulation of AMPK signaling weakens the ability of ageing chondrocytes to resist mitochondrial dysfunction and oxidative stress, thereby accelerating the development of osteoarthritis (Petursson et al., 2013; Matsuzaki et al., 2014; Zhao X. et al., 2014). In subchondral bone, SIRT inhibits the interaction between FoxOs and β-catenin, thereby supporting the wingless-related integration site (Wnt)/β-catenin and T-cell factor (TCF)/lymphoid-enhancing factor (Lef) family-mediated transcription and promoting osteogenesis (Iyer et al., 2014). Furthermore, SIRT suppresses transforming growth factor-β1 (TGF-β1) expression and decreases sclerostin (SOST) levels, thereby stopping the abnormal activation of Wnt signaling and improving dysregulated bone mineralization and sclerosis in chondrocytes (Abed et al., 2014).

### 3.3 Autophagy

**FIGURE 5:**
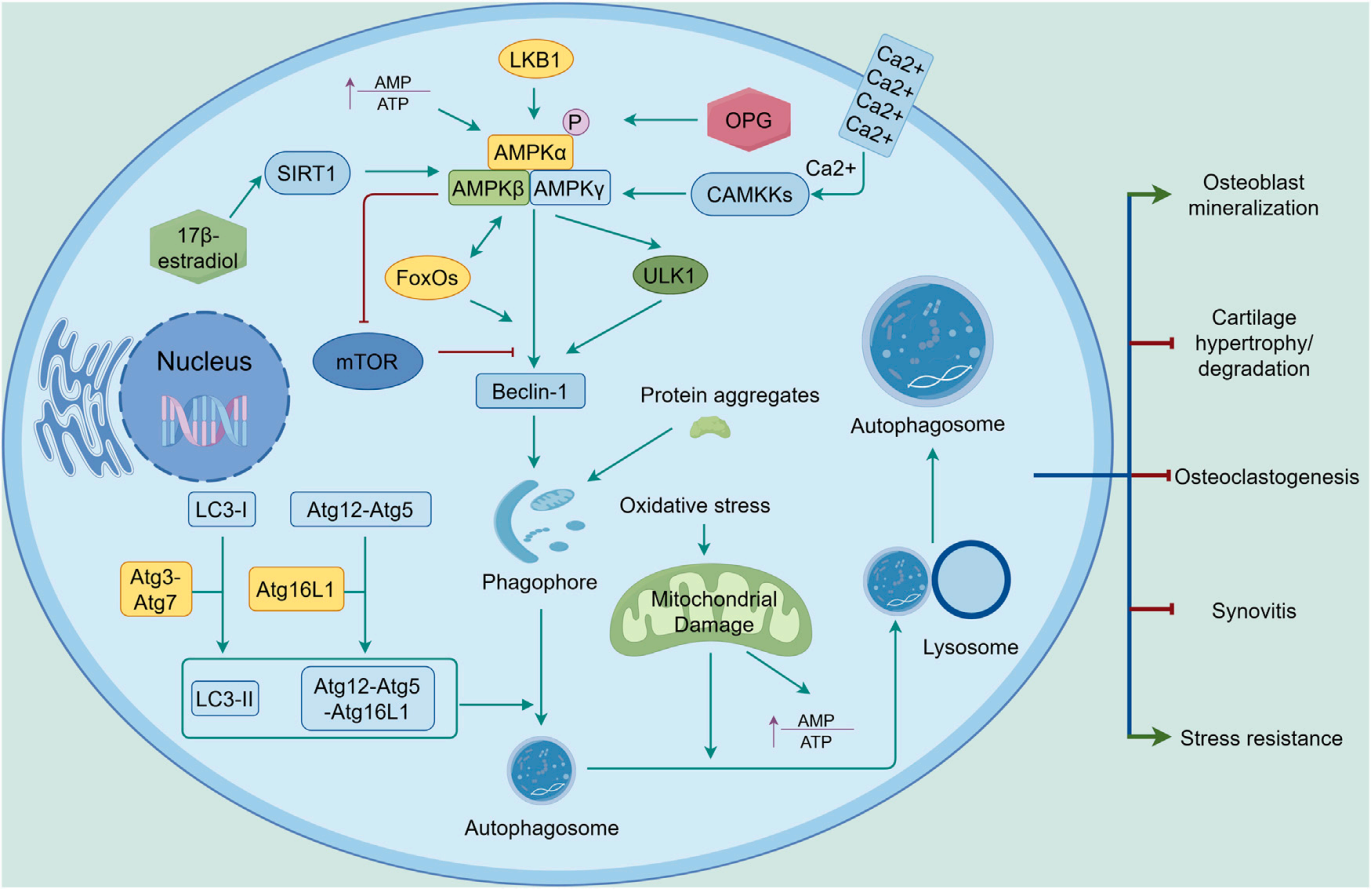
AMPK-mediated autophagy in the joint. Upon activation by increased AMP-to-ATP ratio, LKB1, CAMKKs, or OPG, AMPK signaling promotes autophagy through upregulation of Beclin-1, with ULK1, SIRT1, FoxOs, and mTOR playing key roles in the process. With the assistance of Atg3 and Atg7, LC3-I is converted to LC3-II. The Atg12-Atg5 complex binds to Atg16L1 to form the Atg12-Atg5-Atg16L1 complex. Through the involvement of LC3-II and the Atg12-Atg5-Atg16L1 complex, the phagophore matures into an autophagosome. Damaged mitochondria resulting from oxidative stress are engulfed by the autophagosome and subsequently degraded by lysosomes. Autophagy exerts its effects in joints by inhibiting cartilage degradation, synovitis, and osteoclastogenesis, while enhancing stress resistance and osteoblast mineralization. The green line indicates promotion, and the red line indicates suppression. ↑ signifies an increase.

Autophagy, a cell turnover of cytosolic components, long-lived proteins, or damaged organelles, is able to relieve cell damage and maintain cells with a normal phenotype (Almonte-Becerril et al., 2010). Autophagy also participates in bone homeostasis. For osteoblasts, autophagy is involved in their mineralization to maintain bone mass and protects them from evaluated stress, during which autophagy-related proteins such as autophagy-related 7 (Atg7), Atg5, microtubule-associated protein 1 light chain 3 (LC3-I and LC3-Ⅱ) exert their effect (Nollet et al., 2014). For osteoclasts, Beclin-1(also known as Atg6), Atg5, Atg7, Atg4B, LC3-I and LC3-Ⅱ are indispensable in their differentiation, maturation and osteoclast-mediated bone resorption (DeSelm et al., 2011). Some studies have indicated that AMPK signaling directly or indirectly regulates autophagy, thus helping the balance between catabolic and anabolic factors in joints (Figure 5). For example, activation of AMPK inhibits the mammalian target of rapamycin (mTOR), a protein that directly inhibits autophagy when activated, thereby initiating a protective autophagic program during biomechanical load and preventing articular cartilage degradation and synovial fibrosis (Zhang Y. et al., 2015). This inhibition is also found in T cell-mediated inflammatory responses, which prevents the progression of synovitis (Wen et al., 2019). Further studies have demonstrated that mTOR signaling was crucial in osteoclast maturation, while AMPK activation significantly suppressed osteoclast genesis and promoted osteogenic differentiation by inhibiting mTOR signaling (Indo et al., 2013; She et al., 2014). Osteoprotegerin (OPG) is another important factor in stopping osteoclast activation and promoting osteoclast apoptosis to maintain bone metabolic balance via competitive inhibition of RANK/RANKL connection. AMPK/mTOR signaling is involved in this process (Udagawa et al., 2000; Tong et al., 2020). Furthermore, AMPK phosphorylation directly activates the autophagy-initiating kinase, unc-51-like autophagy activating kinase 1(ULK1) (Kim J. et al., 2011). Under mechanical stress, AMPK activation promotes osteoblast differentiation and bone regeneration via ULK1-mediated autophagy, resulting in increased expression levels of Atg7, LC3B-I and LC3B-II (Zhang S. et al., 2022). FOXOs also synergize with AMPK to activate autophagy by binding to the genes and maintain cellular homeostasis in response to environmental stress (Eijkelenboom and Burgering, 2013). This axis exerts its effects through Ca^2+^ signal transduction in response to mechanical stimulation (Dai et al., 2017). Hormones are another factor influencing AMPK signaling-mediated autophagy. Specifically, 17β-estradiol can upregulate SIRT1 to promote autophagy and inhibit apoptosis in osteoblasts via AMPK/FoxO3A signaling. However, during estrogen withdrawal, osteoblasts secrete osteonectin (Sparc), which downregulates the expression of AMPK/FoxO3A in chondrocytes and promotes their hypertrophy and degeneration, ultimately leading to osteoarthritis (Jiang et al., 2023). It should be noted that AMPK-mediated autophagy plays a role in metabolic processes (Steinberg and Carling, 2019). Dysregulation of autophagy can lead to metabolic diseases such as obesity, which can exacerbate joint damage (see part 3.1) and directly impair the normal function of joints (Wang B. et al., 2021; Cai et al., 2018). Furthermore, autophagy declines in ageing organs, including joints, as AMPK activity decreases with age. The relationship between this decline in autophagy and AMPK remains to be explored (Kim Y. A. et al., 2013).

### 3.4 Anti-inflammation and inflammation

**FIGURE 6:**
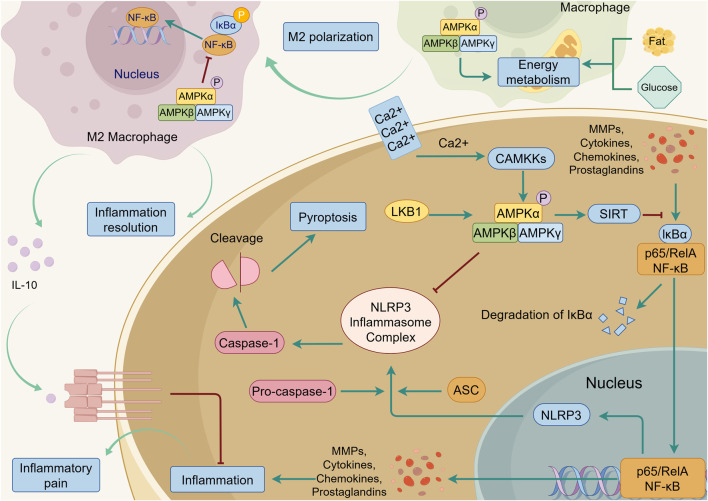
AMPK-mediated anti-inflammation in the joint. Upon activation by LKB1 and CAMKKs, AMPK inhibits NF-κB signaling triggered by MMPs, cytokines, chemokines, and prostaglandins, and suppresses the secretion of these inflammatory factors. NF-κB signaling promotes the assembly of nod-like receptor protein 3 (NLRP3), ASC, and pro-caspase-1 into the NLRP3 inflammasome complex. This complex activates caspase-1, which subsequently induces pyroptosis. AMPK signaling can effectively inhibit this process. In macrophage, AMPK promotes M2 polarization by regulating energy metabolism of fat and glucose. The M2 macrophage can secrete anti-inflammatory factors such as Interleukin-10 (IL-10), which suppresses inflammation and alleviates Inflammatory pain. The green line indicates promotion, and the red line indicates suppression.

OA is associated with ageing joint tissue, which often has low-grade inflammation. This inflammation may be related to the decrease in AMPK activity that occurs with ageing (Petursson et al., 2013; Lotz and Loeser, 2012). Inflammation resolution is essential for the return to tissue homeostasis after an inflammation, however, the failure of inflammation resolution leading to chronic inflammation, impaired repair, and eventually tissue injury is common in OA. Normally, AMPK alleviates inflammation and inhibits abnormal cell death after activation, while macrophages are the key in inflammation resolution regulated by AMPK (Newman et al., 2021; Salminen et al., 2011) (Figure 6). AMPKα1 is the predominant AMPKα isoform expressed in macrophages and deletion of the AMPK β1 subunit and AMPK α1 reduces the function of macrophages, resulting in disorders of fatty and glucose metabolism and inflammation (Caratti et al., 2023; Galic et al., 2011; Sag et al., 2008). By regulating energy metabolism such as glycolysis/oxidative phosphorylation and fatty acid synthesis (FAS)/ fatty acid oxidation (FAO) in macrophage, AMPK exerts its protective effect in inflammation resolution by inducing M2 polarization, which is an anti-inflammatory type of macrophages with the ability to secrete anti-inflammation factors such as Interleukin-10 (IL-10) (Ko et al., 2023; Park et al., 2017; Sag et al., 2008). This regulation is important for maintaining metabolic homeostasis such as inhibiting insulin resistance caused by inflammation, avoiding the metabolic disorder-inflammation cycle and alleviating inflammatory pain (Yang Z. et al., 2010; Russe et al., 2013). In addition, AMPK signaling is able to downregulate the inflammatory signaling directly. In articular cartilage, activation of AMPK attenuates inflammation in chondrocytes stimulated by interleukin-1beta (IL-1β) and tumour necrosis factor-alpha (TNF-α) to maintain the synthesis of ECM components such as proteoglycan (PG), inhibit nuclear factor-kappa B(NF-κB) signaling pathway, and decrease matrix metalloproteinases (MMPs) levels (Terkeltaub et al., 2011). Conversely, in chondrocytes knocked down AMPKα or LKB1, after being induced by IL-1β and TNF-α, release of MMPs and catabolic responses are unregulated, accelerating OA progression (Petursson et al., 2013; Zhou S. et al., 2017). Accumulated inflammation induces pyroptosis, a form of programmed cell death that differs from apoptosis, leading to injury. Inflammasome NF-κB signaling and nod-like receptor protein 3 (NLRP3) take crucial participate in inflammation and pyroptosis, while the AMPK signaling pathway suppresses the production of NLRP3 inflammasome (ASC, Caspase-1, IL-1β, NLRP3, and cleaved Caspase-1) in OA chondrocytes, thus inhibiting pyroptosis and protecting chondrocytes from damage (Chen Y. et al., 2022). In synovium, activated by AMPK phosphorylation, SIRT1 deacetylates RelA/p65 component of the NF-κB complex, and finally inactivates NF-κB signaling. Conversely, NF-κB downregulates SIRT1 activity and accelerates the inflammation (Kauppinen et al., 2013). Activated by extracellular Ca^2+^ influx mediating CaMKKβ, AMPK signaling is also involved in joint mobilization, which suppresses the inflammatory response in synovium induced by TNF-α under mechanical force (Kunanusornchai et al., 2016a). Different from the pro-inflammatory effect shown in part 3.1, most studies have demonstrated the excellent anti-inflammatory properties of AMPK. There are several possible reasons for the conflicting research conclusions: Firstly, since AMPK is the center of energy metabolism and inflammation requires a large amount of energy, the production and activation of AMPK increase accordingly. Secondly, AMPK may exhibit anti-inflammatory and pro-inflammatory effects in different cells. For example, pro-inflammatory effects regulated by AMPK activation are more prevalent in synovial fibroblasts and chondrocytes, whereas in macrophages and other cells, AMPK activation exhibits anti-inflammatory effects. Thirdly, as most literature discusses the anti-inflammatory functions of AMPK, we suspect that enhanced AMPK expression is due to its anti-inflammatory effects in inflammatory processes. Of course, the underlying mechanisms need to be further explained.

## 4 Potential therapies targeting AMPK signaling in OA

The important role of AMPK in maintaining tissue homeostasis within joints highlights its potential as a therapeutic target for OA. Experimental studies have demonstrated that enhancing AMPK signaling can alleviate OA progression, and therapies targeting AMPK signaling are under development.

### 4.1 Metformin

Some researchers have shown that Metformin exerts its effect on glucose and lipid metabolism by promoting AMPK activation (Zhou G. et al., 2001). In a series of experiments, metformin is able to protect cartilage, relieve synovial inflammatory reactions, and thus alleviate the development and OA progression (He et al., 2022). For example, Jun Li et al. find that metformin delays OA progression and attenuates OA pain in wild-type mice; however, this protective effect is completely absent in mice with knockdown of AMPK α1. Furthermore, they confirm that metformin alleviates OA progression in non-human primates, which identifies the effectiveness of metformin on OA pathogenesis (Li J. et al., 2020). Following the activation of AMPK signaling by metformin, its downstream SIRT1 signaling and acetyl-CoA carboxylase (ACC) signaling are enhanced while β-catenin signaling is suppressed, which contributes to autophagy in chondrocytes, preventing the synthesis, accumulation and deposition of cholesterol, and inhibiting ferroptosis (Zhu et al., 2022; Wang C. Z. et al., 2020; Xing et al., 2022; Zou et al., 2025). Metformin also attenuates the inflammatory response of synovitis and increases the levels of hyaluronan and proteoglycan link protein 1 (HAPLN1) through the activation of AMPK signaling, which helps to improve synovium (Chen Y. et al., 2020).

### 4.2 Direct AMPK activator under development

Given the important role of AMPK in metabolic processes, many studies have attempted to develop its direct activators to treat metabolic diseases. Several small molecules developed as allosteric activators of AMPK appear to bind to the ‘allosteric drug and metabolite’ (ADaM) site, which connects the regulatory β subunit and the catalytic α subunit (Steinberg and Carling, 2019). Furthermore, some activators can suppress the dephosphorylation of pAMPK, thereby maintaining AMPK activity. These AMPK activators have been shown to be highly effective in both animal and human studies.

#### 4.2.1 PXL770

PXL770 is an orally bioavailable thienopyridone small molecule that increases AMPK activity by both allosteric activation and protection from dephosphorylation. Study shows that it has the ability to reverse hyperglycemia, enhances insulin sensitivity, and ameliorates other metabolic disorders in rats with nonalcoholic steatohepatitis (Gluais-Dagorn et al., 2022). In a randomized, double-blind four-week trial, PXL770 is shown to improve metabolic disorders in overweight/obese patients with non-alcoholic fatty liver disease (NAFLD) and insulin resistance (Fouqueray et al., 2021).

#### 4.2.2 O304

With the ability to suppress dephosphorylation of pAMPK, O304 is found to increase glucose uptake in skeletal muscle, reduce β cell stress, and promote β cell rest in diet-induced obese (DIO) mice. A subsequent clinical trial demonstrated that O304 improves glucose homeostasis in patients with type 2 diabetes (Steneberg et al., 2018).

#### 4.2.3 PF-739

PF-739 is a benzimidazole derivative that binds to and activates all AMPK heterotrimers with similar potency via the ADaM site. PF-739 has been shown to decrease plasma glucose levels in DIO mice and cynomolgus monkeys by activating AMPK signaling in skeletal muscle (Cokorinos et al., 2017).

#### 4.2.4 PF-06409577

PF-06409577-mediated activation of AMPK can inhibit the pathways involved in *de novo* lipid and cholesterol synthesis, thereby reducing hepatic lipids and the expression of markers of hepatic fibrosis. This lowers hepatic and systemic lipid and cholesterol levels in mice and cynomolgus monkeys with NAFLD (Esquejo et al., 2018).

#### 4.2.5 MK-8722

As an effective activator of β complexes, MK-8722 is developed to improve glucose homeostasis in dysmetabolic and diabetic rodents and rhesus monkeys by inducing glucose uptake and glycogen synthesis in skeletal muscle (Myers et al., 2017).

Although different AMPK activators are emerging, their appropriate targeted disease and potential adverse effects need to be further confirmed. It deserves more exploration into this area.

### 4.3 Natural medicine

A series of studies have explored the protective effects of traditional medicine in relieving osteoarthritis from anti-inflammation to anti-oxidative stress. Many of these studies have revealed AMPK activation as a key underlying mechanism (Table 1).

**TABLE 1:** Natural medicines that target AMPK signaling in the treatment of OA.

Therapy	Research subjects	Model	Year	References	Effects
Geniposide	Sprague-Dawley (SD) rats. C28/I2 human chondrocyte cell line	Intra-articular injection of monosodium iodoacetate (MIA). IL-1β-induced chondrocyte injury	2023	Huang et al. (2023)	Promotion of chondrocyte autophagy in OA chondrocyte through activation of GLP-1R/AMPK/mTOR pathway
Sinensetin	10-week-old male C57BL/6 mice. Primary chondrocytes isolated from mice	Destabilization of the medial meniscus (DMM). Tert-butyl hydroperoxide (TBHP)-induced injury	2021	Zhou W. et al. (2021)	Promotion of chondrocyte autophagy in OA chondrocyte through activation of AMPK/mTOR pathway
Resveratrol	10-week-old male C57BL/6 mice	DMM.	2017	Qin et al. (2017)	Promotion of chondrocyte autophagy in OA chondrocyte through activation of AMPK/mTOR pathway
Osthole	SD rats. Primary chondrocytes isolated from rats	Huth method. IL-1β-induced chondrocyte injury	2022	Ma T. et al. (2022)	Promotion of chondrocyte autophagy in OA chondrocyte through activation of AMPK/ULK1 pathway
Harpagide	SD rats. Primary chondrocytes isolated from rats	Anterior cruciate ligament transection (ACLT). TNF-α-induced chondrocyte injury	2024	Xu et al. (2024)	Inhibition of cartilage degeneration and inflammation through activation of AMPK signaling
Hesperetin	SD rats. Primary chondrocytes isolated from rats	ACLT. TNF-α-induced chondrocyte injury	2021	Wu et al. (2021)	Inhibition of cartilage degeneration and inflammation through activation of AMPK signaling
Chitosan oligosaccharide	Three-month-old New Zealand White rabbits. Human synoviocytes from OA patients. Primary chondrocytes isolated from New Zealand White rabbits	ACLT. TNF-α-induced synovitis	2016	Kunanusornchai et al. (2016b)	Inhibition of synovitis through activation of AMPK signaling
Berberine	12-week-old male C57bl/6 mice. Primary chondrocytes isolated from mice. Human knee chondrocytes	DMM. IL-1β-induced chondrocyte injury	2022	Li J. et al. (2022)	Inhibition of cartilage degeneration and inflammation through activation of AMPK/SIRT1 signaling
Bilobalide	7-week-old male C57bl/6 mice. Human knee chondrocytes	DMM. IL-1β-induced chondrocyte injury	2022	Zhao Z. et al. (2022)	Inhibition of cartilage degeneration and inflammation through activation of AMPK/SIRT1 signaling
	SD rats. ATDC5 cells	ACLT. IL-1β-induced chondrocyte injury	2022	Ma et al. (2022)	Inhibition of cartilage degeneration and inflammation through activation of AMPK/SIRT1/mTOR signaling
Safflower yellow	SD rats. Primary rat chondrocytes	ACLT. TNF-α-induced chondrocyte injury	2020	Wang C. L. et al. (2020)	Inhibition of cartilage degeneration and inflammation through NF-κB/SIRT1/AMPK signaling
Total flavonoids from Rhizoma Drynariae	SD rats. Primary chondrocytes isolated from OA patients	Huth method. IL-1β-induced chondrocyte injury	2023	Chen G. Y. et al. (2023)	Prevention of cartilage degeneration and inflammation through activation of AMPK signaling and inhibition of NF-κB
Xanthohumol	SD rats. Human chondrocytes from OA patients	DMM. Palmitate (PA)-induced chondrocyte injury	2023	Sun W. et al. (2023)	Inhibition of cartilage degeneration, NLRP3 inflammasome, mitochondria dysfunction through activation of AMPK/NF-κB signaling
Quercetin	Male SD rats. Primary chondrocytes isolated from rats	Medial meniscotibial ligament transection. TBHP-induced injury	2019	Feng et al. (2019)	Inhibition of ER stress through AMPK/SIRT1 signaling
	Male SD rats. Primary chondrocytes isolated from rats	DMM.	2018	Qiu et al. (2018)	Inhibition of mitochondrial dysfunction through AMPK/SIRT1 signaling
Baicalein	8-week-old C57BL/6J wild-type mice. Human knee chondrocytes. Primary chondrocytes isolated from mice	DMM. IL-1β-induced chondrocyte injury	2023	Wan et al. (2023)	Inhibition of ferroptosis through AMPK/Nrf2 signaling
Asiatic acid	Male SD rats. Human knee chondrocytes	ACLT.	2020	Liu et al. (2020)	Inhibition of chondrocyte hypertrophy and fibrosis through AMPK/PI3K/AKT signaling

#### 4.3.1 Increased autophagy

Geniposide (GEN, an iridoid glycoside extracted from Eucommia ulmoides Oliv), Sinensetin (Sin, a polymethoxylated flavonoid found in citrus fruits), Resveratrol (a polyphenolic phytoalexin presents in many plants) and Osthole (an ingredient from the root of Freziera biserrate) have been confirmed their abilities to mediate autophagy for maintaining cartilage homeostasis, protecting chondrocytes and ECM from damage, deformation and degeneration, and attenuating OA pain via AMPK/mTOR or AMPK/ULK1 signaling (Huang et al., 2023; Zhou W. et al., 2021; Qin et al., 2017; Ma et al., 2022).

#### 4.3.2 Anti-inflammation

The anti-inflammatory effects of natural medicines have been used to alleviate OA progression. Harpagide (an iridoid glycoside natural molecule from the root of Harpagophytum procumbens var. sublobatum (Engl.)), Hesperetin (a natural flavonoid from the *Citrus L.*) and Chitosan oligosaccharide (COS, an oligomer of d-glucosamine) have been found to suppress the expression of inflammatory cytokines and MMP, thereby protecting cartilage from damage and abnormal proliferation, improving synovitis, inhibiting osteophyte formation and attenuating pain through AMPK signaling (Xu et al., 2024; Wu et al., 2021; Kunanusornchai et al., 2016b). Further investigation revealed that these effects are regulated by the activation of AMPK and its downstream signaling pathways. For example, SIRT1 signaling could be regulated by Berberine (an isoquinoline alkaloid extracted from plants) and Bilobalide (a lactone extracted from *Ginkgo biloba*) (Li J. et al., 2022; Zhao Z. et al., 2022; Ma et al., 2022). NF-κB signaling was inhibited by Safflower yellow (an active ingredient of Carthamus tinctorius L.), total flavonoids from Rhizoma Drynariae (the main active ingredients from the dried rhizome of *Davallia mariesii* T. Moore ex Baker) (Wang C. L. et al., 2020; Chen G. Y. et al., 2023). And NLRP3 inflammasome pathway was suppressed by Xanthohumol (Sun W. et al., 2023).

#### 4.3.3 Oxidative stress

Some medicines have been found to decrease oxidative stress and attenuate OA progression by modulating AMPK signaling and its downstream pathways. For instance, quercetin inhibited ER stress and mitochondrial dysfunction via AMPK/SIRT1 signaling (Feng et al., 2019; Qiu et al., 2018). Baicalein activated AMPK signaling and then induced nuclear factor erythroid 2-related factor 2 (Nrf2), a key regulator of antioxidant responses, to block ferroptosis (Wan et al., 2023). Asiatic acid (a pentacyclic triterpene isolated from *Centella asiatica*) had the ability to reduce hypertrophic and fibrotic differentiation by targeting the AMPK/PI3K/AKT signaling pathway (Liu et al., 2020).

Natural medicines offer promising prospects for the treatment of OA, with some of them showing positive effects in the activation of AMPK signaling. Nonetheless, several issues need to be addressed: First, the composition and interaction of these ingredients of TCM are under exploration. Second, it is hard to make a conclusion that these natural medicines exert their effect by directly activating AMPK since they did not explore the structure of AMPK or the combination of compounds and AMPK after the application. Third, the appropriate dosage range and potential therapeutic relevance are not fully defined and explained. High doses are not beneficial in humans and lack translational value due to concerns about toxicity and bioavailability.

## 5 Discussion

The activation of AMPK signaling offers protection against environmental stress by promoting autophagy and mitochondrial biogenesis. By contrast, disturbances in AMPK signaling resulting from metabolic disorder lead to cellular injury or even death due to oxidative stress, inflammation. The impact of AMPK signaling dysregulation during OA progression highlights the therapeutic potential of targeting AMPK signaling as a promising strategy for OA (Figures 2–6). It should be noted that these mechanisms do not exist in isolation but affect each other. Metabolic disorders are caused by oxidative stress, the inhibition of autophagy, and inflammation. Inflammation aggravates oxidative stress. Oxidative stress leads to the production of inflammatory factors, thereby promoting inflammation. All of these factors inhibit autophagy, which further causes metabolic disorders. AMPK, at the center of energy metabolism, participates in almost every energy-requiring biological process, reflecting its important role in maintaining homeostasis.

However, there are several limitations of targeting AMPK signaling in the treatment of OA. First, although numerous animal studies have confirmed the role of AMPK signaling in maintaining joint homeostasis, there is a lack of clinical trials demonstrating the effectiveness of AMPK-targeted therapy for OA patients. Some human clinical studies have reported the beneficial effect of metformin in OA (Wang Y. et al., 2019). However, a record cohort study enrolling 3217 patients with type 2 diabetes in the United Kingdom found that metformin treatment had no significant effect on the progression of OA (Barnett et al., 2017).

Overall, due to the current limitations in OA management, exploring novel therapies is crucial. In the future, more effort will be put into clarifying the role of AMPK signaling in OA progression and clinical application to protect patients from OA damage.
